# Characterization and evaluation of Greek tomato landraces for productivity and fruit quality traits related to sustainable low-input farming systems

**DOI:** 10.3389/fpls.2022.994530

**Published:** 2022-12-12

**Authors:** R. I. Tagiakas, I. D. Avdikos, A. Goula, K. Koutis, Irini Nianiou-Obeidat, A. G. Mavromatis

**Affiliations:** ^1^ Aristotle University, Lab of Genetics and Plant Breeding, School of Agriculture, Thessaloniki, Greece; ^2^ International Hellenic University, Lab of Horticulture, Thessaloniki, Greece; ^3^ Aristotle University, Lab of Food Engineering and Processing, School of Agriculture, Thessaloniki, Greece; ^4^ Aegilops, Network for Biodiversity, Volos, Greece

**Keywords:** *Solanum lycopersicum*, organic culture, nutritive value, breeding, pure line selection

## Abstract

Tomato is one of the most important horticultural species all over the world, having high level of consumption and employing many people, both in the primary sector (farmers) and in the secondary sector (traders, seed companies and processors). Nowadays, the use of commercial tomato F1 hybrids tends to prevail because of high yield potential and homogeneity of fruits which are often characterized by lack of quality and sensory characteristics. In contrast, tomato landraces have outstanding quality traits, such as high concentration of antioxidants and organoleptic compounds, as well as often include desirable genes in their genome for adaptability, plasticity, response to low-input conditions, and high fruit nutritional value. Thus, they are appropriate material in the use of sustainable agricultural management systems or as gene donors for the development of new type of tomato cultivars suitable for low-input farming systems. The present experimental study refers to 22 Greek tomato landraces and two commercial cultivars (cv. Macedonia and the F1 hybrid Formula) used as controls, which were characterized by phenotypical markers and evaluated under low-input sustainable farming conditions. Specifically, during this research, measurements were taken regarding yield potential (early production, number of fruits per plant, fruit weight, total yield) and fruit quality traits, such as physicochemical characteristics (pH, acidity, and soluble solid components – Brix^ο^) also according to nutritional value (content of ascorbic acid, lycopene, total carotenoids, and total phenolics) of tomato fruits. In the most promising landraces (cv. Milo Chalkidiki, cv. Eratiras, cv. Lotos, cv. Aspros lotos, cv. Pantaroza, cv. Karabola and cv. Kardia Vodiou), having comparable yield and fruit quality traits with commercial cultivars, intrapopulation “Pure line selection” method, under low-input farming conditions was applied for two years. Following this approach, we succeed to determine the level of yield potential and provide information for the nutritive value and utilization of typical tomato landraces, improving their yield and fruit quality traits, following a mild intrapopulation selection under low-input farming conditions. This data pipeline is expected to be of interest for organic farmers and processors of high nutritive tomato products, with low carbon footprint for the environment.

## Introduction

Tomato (*S. lycopersicum* L.) is among the most widely grown and consumed vegetables in the world, with more than 4000 registered varieties only in the European Union (FAO, 2018; Plant variety database https://ec.europa.eu/food/plant). Aside from its socio-economic importance, tomato has become a model species for fleshy fruits, because of its agronomic and genetic features, and particularly as a rich plant source of bioactive compounds like carotenoids, vitamins, and minerals (Bergougnoux, 2014; Schwarz et al., 2014).

Given the predicted rise in world population, fruits and vegetables like tomato are expected to become the main source of secondary metabolites for millions of persons in the near future. Therefore, the greatest challenge in next years will be to increase crop production and fruit quality, reducing simultaneously the inputs and carbon fingerprinting into agroecosystems (Mavromatis et al., 2013).

Of course, the quality term is very wide and may refer to intrinsic and extrinsic characteristics, as well as to preharvest and postharvest stages (Kyriacou and Rouphael, 2018). The synthesis and accumulation of health-promoting metabolites, termed phytochemicals, depend mainly on the genetic material, but also on the agronomic practices and environmental factors, which have an important influence on yield and quality characteristics of fruits (Rouphael et al., 2012; Schreiner et al., 2013). The improvement of nutritional quality by enhancing the contents of bioactive compounds like lycopene has become an important aspect of tomato fruit quality valorization, and it has emerged as a challenge for growers who want to meet the ever-increasing demands of consumers (Hallmann, 2012; Mavromatis et al., 2013).

Nowadays, other priorities for the international society are the energy equilibrium in agroecosystems and carbon fingerprinting into the final product. For these reasons, low-input cultivation systems support these demands and might be positively influenced in an environmentally friendly way, for the production of more healthy products, ensuring the level of biodiversity in agroecosystems (Dennis et al., 2017).

In the last 80 years, monoculture and restriction in the use of landraces and indigenous species have led to the genetic erosion phenomena observed in many crops and horticultural species. A significant part of agricultural biodiversity consists of landraces maintained by farmers (Villa et al., 2005). The recovery of locally adapted landraces could play a very important role in avoiding, at least partially, production losses and simultaneously improving fruit quality (Massaretto et al., 2018). Landraces are dynamic populations of cultivated plants with historical origins, distinct identities, and lack of formal crop improvement (Casañas et al., 2017). Landraces are usually associated with traditional farming systems and have evolved under natural and farmers’ selection in low-input agricultural systems (Terzopoulos and Bebeli, 2008; Mavromatis et al., 2013). Also, this genetic material is typically characterized by high stress tolerance and local adaptability (Newton et al., 2011; Hawtin et al., 1996; Andreakis et al., 2004; Acciarri et al., 2010; Digilio et al., 2010).

Alternatively, local landraces selected for centuries under the severe conditions of the Mediterranean region and countries like Greece may also be a very suitable genetic pool to improve tomato crop tolerance to the drier or to low-input conditions and into crop adaptation, giving specific quality traits (Hawtin et al., 1996; Hoisington et al., 1999; Huang et al., 2012).

Furthermore, landraces are gaining increasing attention considering their value for niche markets, yield stability in low-input agricultural systems, and the growing popularity of sustainable farming systems like organic culture (Fernie et al., 2006; Berg, 2009). Typical agri-food products originated from landraces are those distinct from others available on the market, because they belong to the historical memory of their origin and production areas, possessing superior physicochemical, sensory or dietary characteristics (Mavromatis et al., 2013).

Thus, it is necessary to increase our knowledge on the response of those local landraces to low-input conditions, in order determine their possible role in the near future pedoclimatic conditions. The objective of this study was: (a) to provide information for the value and utilization of typical tomato landraces from the Mediterranean region, especially from Greece, as related to physicochemical and nutritional traits in relation to their response in low-input farming conditions, and secondly, (b) to improve distinctly the yield potential and fruit quality traits of these landraces according to farmer’s and consumer’s preferences, following a mild intrapopulation selection under low-input farming conditions.

## Materials and methods

### Genetic material and growing conditions

The present study was conducted on the farm of Faculty of Agriculture, Aristotle University of Thessaloniki. More specifically, to carry out this experiment, 22 tomato landraces (**Table 1**), from different geographical areas of Greece, were used. Also, two commercial tomato cultivars, (cv. Macedonia and cv. Formula), were used as controls. “Macedonia” is a pure line cultivar, developed by the Agricultural Research Center of Northern Greece (ARCNG), indeterminate and well adapted to both glass-covered and open-field cropping conditions of the Mediterranean region, and it is characterized by preferable physicochemical and sensory properties with attractive and tasty fresh fruits (Avdikos et al., 2021b). “Formula F_1_” is a Golden West commercial F1 hybrid, characterized by high yield and standard quality traits under greenhouse and open-field conventional cropping conditions.

**Table 1:** Genetic material (tomato landraces) used in the experiment (first phase).

Experiment code	Name of tomato landrace	Seed bank	Origin
*1*	Filia Lesvou	Aegilops*	Island of Lesvos
*2*	Atheras	Aegilops	-
*3*	Agion Oros	Aegilops	Saint Athos
*4*	Milo Chalkidiki	A.U.Th.**	Chalkidiki
*5*	Souvritiki Evrou	A.U.Th.	Region of Evros
*6*	Boulgariki	Aegilops	Region of Macedonia
*7*	Macedonia (Control I)	ARCNG***	Region of Macedonia
*8*	Milo Corfu	A.U.Th.	Island of Corfu
*9*	Milo Cephalonia	Aegilops	Island of Cephalonia
*10*	Imvros	A.U.Th.	Island of Imbros
*11*	Trikala Imathias	A.U.Th.	Trikala Imathias
*12*	Formula F1 (Control ll)	Golden West	Golden West company
*13*	Eratiras	Aegilops	Eratira – Kozani
*14*	Lotos	A.U.Th.	Volos – Thessaly
*15*	Nikoulas	Aegilops	-
*16*	Evrou	A.U.Th.	Region of Evros
*17*	Feneou	Aegilops	Feneos/Region of Peloponnese
*18*	Aspros lotos	A.U.Th.	Volos – Thessaly
*19*	Pantaroza	A.U.Th.	Kefalonia Island
*20*	Karabola	A.U.Th.	Region of Macedonia
*21*	Kardia vodiou	A.U.Th.	Region of Epirus
*22*	Takas	A.U.Th.	-
*23*	Pastra	A.U.Th.	Athens
*24*	Milo Serron	A.U.Th.	Serres – Region of Macedonia

* Aegilops Network for Biodiversity and Ecology in Agriculture – a network of eco farmers in Greece.

** Laboratory of Genetics and Plant Breeding of Agriculture Faculty of the Aristotle University of Thessaloniki.

*** Agricultural Research Center of Northern Greece.

**Figure 1:**
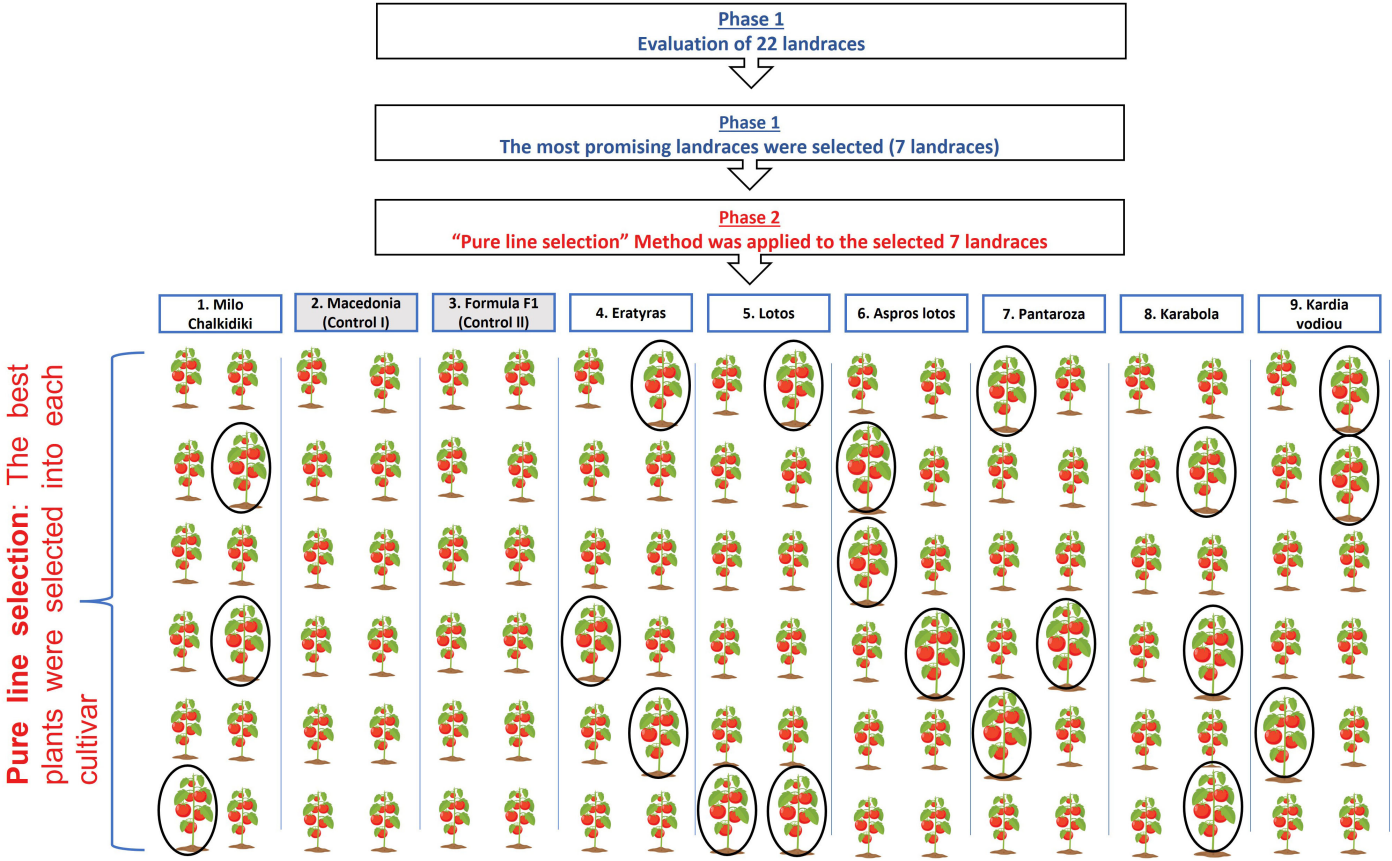
Schematic presentation of Experimentation: Two Phases were followed: the (I) Evaluation Phase and the (II) Selection Phase.

First of all, at the first phase, we evaluated 22 landraces for 2 years (**Figure 1**) using randomized complete block design (RCBD) for experimentation. In each case, three replications were applied, consisting of 12 plants per cultivar. The experiment was carried out in open field conditions under a low-input cultivation system (lower inputs comparing with to conventional standard used by the farmers) to reduce the carbon footprint. The experimental field was shaded using a shading net to reduce the intensity of solar radiation (30%) during June, July, and August. In greater detail, the low-input conditions include half quantities of inputs related to fertilizers, irrigation, and pesticide applications, in comparison to the conventional farmer’s program. Organic fertilizers and pesticides permitted in organic sustainable management were used. More specifically, organic fertilization with manure was applied, in a quantity of 3 t/ha (dry weight) and there were no chemical pesticides used for plant protection purposes, but only those compatible with the principles of organic farming (Copper hydroxide, Sulphur, *Bacillus thuringiensis*, etc.).

### Description of morphological traits

To detect the phenotypic variability, 14 morphological characteristics were measured, according to the guideline of International Union for the Protection of New Varieties of Plants (UPOV, 2011). More specifically, these characteristics were obtained on an individual plant basis and are presented in **Table S1**.

### Analysis of yield components

Total yield was measured on individual plant basis. The total yield was formed by four harvesting dates (70, 85, 102, and 148 days after transplanting). The first three harvests determined the early yield component (70 until 102 days after transplanting, depends on cultivar) and total yield potential (until 148 days after transplanting). Each harvest was carried out by hand, at the red ripe stage of fruits (OCDE/OECD, 1992). The yield components measured were the number of fruits per plant and the mean fruit weight, regarding to the early yield and to the total yield potential.

### Analysis of fruit quality and nutritional value traits

For the analysis of the quality and nutritional value traits, three red ripe fruits per cultivar were harvested at Red Ripe Stage, the first and second fruits of the third inflorescence of each cultivar *per* replication. The quality traits of fruits that were evaluated included the calculation of total soluble solid content (Brix), pH, and acidity estimation of tomato juice (% citric acid). The nutritional value traits included the composition of tomato fruits in Ascorbic acid (vitamin C), lycopene, carotenoids, and total phenols. The analysis of these characteristics was evaluated in the Laboratory of Food Engineering and Processing of Agriculture Faculty A.U.Th.

To determine the total soluble solids, tomatoes were chopped into pieces with a knife and were ground into rough pulp using a StarMix blender (Model H.1, Starmix S.p.A., Schio, Italy). Subsequently, rough pulp was passed through a finisher (0.5 mm screen) to get a thin tomato pulp. The pulp was stored in a refrigerator at 5°C overnight before subsequent analysis. Specifically, approximately 10 g of pulp was filtered through filter paper. This filtrate was placed on a glass refractometer to measure soluble solids. The total soluble solid (TSS) was determined by a Reichert Mark II plus refractrometer (Reichert Inc., United States).

To measure the pH, the fruits were cut into four quadrants and the two diagonals from each fruit were selected. The six parts of the fruit were homogenized in a mixer. The solution was then filtered using paper, and about 10 ml of the filtrate was used to measure the pH with a digital pH meter (WTW pH meter, inoLab, Weilheim, Germany), which has been standardized with buffers at pH 4 and 7.

The acidity in tomato pulp is expressed as a percentage of citric acid. The estimation of the acidity of tomato pulp was conducted with the method of neutralization reaction. The percentage of citric acid contained was calculated as follows (Goose, 1964):


$$\%\text{citric acid=}\frac{7\cdot \text{k}\cdot \text{V}}{100\cdot \epsilon }$$

where: k: tomato pulp dilution factor (-)

V: volume of sodium hydroxide solution for titration (ml)

ϵ: specific gravity of tomato pulp dilution (-)

The volumetric method was used to determine the ascorbic acid content of tomato pulp (Goose, 1964). The ascorbic acid contained in the sample is calculated as follows (Goose, 1964):


$$\text{Ascorbic acid }(\text{mg}/100\text{g pulp})=\frac{80\cdot {\text{V}}_{1}\cdot {\text{V}}_{\alpha }}{\text{m}\cdot {\text{V}}_{2}\cdot {\text{V}}_{\delta }}$$

where: V_1_: volume of dye solution for filtrate titration (ml)

V_2_: volume of dye solution for the titration of the standard solution ascorbic acid (ml)

V_α_: volume of diluted pulp (ml)

V_δ_: volume of filtrate titrated (ml)

m: volume of filtrate titrated (ml)

To determine the lycopene content of tomato pulp, it was detected spectrophotometrically on extracts in a mixture of acetone-petroleum ether in triplicate at 505 nm (Gould & Gould, 1988) using an Ultrospec II UV/Vis spectrophotometer (LKB Biochrom, Cambridge, UK). The lycopene was quantified by using a standard curve of purified lycopene dissolved in petroleum ether in concentration ranging from 0.20 to 56.25 μg/ml (Goula et al., 2006).

The fruit content of total carotenoids was measured by the extraction method based on Lachman et al. (2003), where the sample (0.125 g) is extracted by an instant ultrasound device (VCX-130, Sonics and Materials, Danbury, CT, USA) with 15 ml of acetone for 60 min.

The extract is filtered and transferred to a 50 ml volumetric flask, then filled with acetone until the volume reaches 25 ml. The absorbance is measured at 662, 645, and 470 nm. The concentration (μg/ml of extract) is calculated as follows:


$$C_{a}=11.75A_{662}-2.35A_{645}$$

$$C_{b}=18.61A_{662645}-3.96A_{662}$$

$$C_{carotenoids}=(1000A_{470}-2.27C_{a}-81.4C_{b})/227$$

The determination of total phenolic compounds was performed by the Folin-Ciocalteu method (Chun et al., 2005). This method is based on the ability of phenolic compounds to reduce phosphomolybdene and phospholobramic acid compounds contained in the Folin-Ciocalteu reagent. The concentration of total phenolic compounds is determined by the absorbance value at 760 nm. Total phenolic compounds determined by the Folin-Ciocalteu method were expressed in Gallic Acid Equivalents (GAE) (mg/ml). For this analysis, an instant ultrasound device (VCX-130, Sonics and Materials, Danbury, CT, USA) and an Ultrospec II UV/Vis spectrophotometer (LKB Biochrom, Cambridge, UK) were used.

### Breeding approach using the pure line selection method

At the second phase of our research (**Figure 1**), intrapopulation “pure line selection” method under low-input farming conditions was applied for 2 years, in the most promising seven landraces, having comparable yield and fruit quality traits with commercial cultivars, used as controls (**Table 2**). This mild breeding approach was applied for these landraces in open field conditions under organic conditions (low-input cultivation system). Following this approach, we tried to determine the level of yield potential of these landraces, improving simultaneously their yield and fruit quality traits. To achieve our purpose, we applied selection following the three most important yield components (number of fruits per plant, weight of fruits per plant and total yield of commercial fruits per plant) and two parameters related to the most important bioactive compounds (lycopene concentration and total carotenoids per fruit) referring to tomato’s nutritional value.

**Table 2:** Genetic material (tomato landraces) used in the second phase of this experiment.

Experiment code	Name of tomato landrace	Seed bank	Origin
*1*	Milo Chalkidiki	A.U.Th.*	Chalkidiki
*2*	Macedonia (Control I)	ARCNG**	Region of Macedonia
*3*	Formula F1 (Control ll)	Golden West	Golden West company
*4*	Eratiras	Aegilops***	Eratira – Kozani
*5*	Lotos	A.U.Th.	Volos -Thessaly
*6*	Aspros lotos	A.U.Th.	Volos -Thessaly
*7*	Pantaroza	A.U.Th.	Kefalonia Island
*8*	Karabola	A.U.Th.	Region of Macedonia
*9*	Kardia vodiou	A.U.Th.	Region of Epirus

* Aegilops Network for Biodiversity and Ecology in Agriculture – a network of eco farmers in Greece.

** Laboratory of Genetics and Plant Breeding of Agriculture Faculty of the Aristotle University of Thessaloniki.

*** Agricultural Research Center of Northern Greece.

### Statistical analysis

Statistical analysis was performed using analysis of variance (ANOVA) for randomized complete block design (RCBD) (with the genotypes as fixed factors and the blocks as random factors). The comparison among means was made using the Duncan multiple range test at 5% probability and significance level (0.95) (Steel and Torrie, 1980). The comparison of the genetic materials (superiority or deficiency) regarding the yield components and the quality traits was estimated in relation to the control cultivar “Macedonia” during a 2-year experimentation. The stability of performance per cultivar was defined using the standardized mean (X/s) which is expressed by the quotient of mean by standard deviation (Fasoula and Fasoula, 2002; Avdikos et al., 2021b). Thus, according to Fasoula and Fasoula (2002), the cultivars combining the largest value of Mean Yield (X) and the largest value for (X/s) are characterized as the most productive and more stable across the environment. The above statistical analyses were performed using the statistical software program SPSS (ver. 24, SPSS Inc., Chicago, IL, USA).

To assess the genetic distances among all tomato landraces used into this experiment, hierarchical analysis, using the results for productivity as related to total yield, number, and weight of fruits *per* plant and the content in nutrition value traits of fruits (lycopene and carotenoids), was used. Measurements from morphological and quality characteristics were analyzed as well using multivariate cluster analysis. This analysis was used for the construction of a dendrogram of HCA, using the unweighted pair group method, with arithmetic mean analysis and the square Euclidean distance as a measure of the genetic distances (Wishart, 1987; Franco et al., 1997).

## Results

### Description of morphological traits

Based on the results of the phenotypic evaluation (**Table 3**), the landrace “Aspros lotos” exhibited the highest height (154.58 cm), in contrast to “Imvros”, which ranked last (77.25 cm). Generally, the average total height was 124.40 cm. Similarly, the landraces “Filia Lesvou” (150.81 cm), “Kardia Vodiou” (150.00 cm), and “Milo of Corfu” (149.22 cm) exhibited an adequate height ability of plants without significant differences with Aspros Lotos.

**Table 3:** Average plant’s morphological traits (height of plant, number of inflorescences per plant, height of fourth inflorescence and length of internode) according to the UPOV guideline (2011).

Plants’ morphological characteristics				
Landraces	Plant: height (cm)	Inflorescence: number of inflorescences	Plant: height of fourth inflorescence (cm)	Stem: length of internodes (cm)
**1. **Filia Lesvou	150.81 ab*	6.89 abcd	80.50 abcd	6.21 abc
**2. **Atheras	129.42 abcde	6.92 abcd	78.33 bcd	6.36 ab
**3. **Agion Oros	133.11 abcd	7.56 ab	80.55 abcd	5.74 bcd
**4. **Milo Chalkidiki	117.83 cde	6.00 bcde	76.92 cde	5.72 bcd
**5. **Souvritiki Evrou	109.97 def	5.69 bcde	82.95 abcd	5.84 bcd
**6. **Boulgariki	107.00 def	4.89 de	81.44 abcd	5.41 bcde
**7. **Macedonia	139.08 abcd	6.83 abcd	84.08 abcd	6.08 abcd
**8. **Milo Corfu	149.22 abc	6.45 abcd	92.34 abc	6.85 a
**9. **Milo Cephalonia	136.39 abcd	7.64 ab	77.86 cde	5.30 cde
**10. **Imvros	77.25 g	4.25 e	74.50 de	4.67 e
**11. **Trikala Imathias	118.08 cde	6.92 abcd	75.33 de	5.28 cde
**12. **Formula F1	130.25 abcd	7.67 ab	76.92 cde	5.33 cde
**13. **Eratiras	113.08 de	6.33 abcd	74.67 de	5.25 cde
**14. **Lotos	132.75 abcd	7.17 abc	81.50 abcd	5.47 bcde
**15. **Nikoulas	122.86 abcde	5.25 cde	93.89 a	5.11 de
**16. **Evrou	126.64 abcde	6.53 abcd	79.58 abcd	5.72 bcd
**17. **Feneou	116.00 de	6.63 abcd	75.75 de	5.33 cde
**18. **Aspros Lotos	154.58 a	8.42 a	76.67 de	5.89 bcd
**19. **Pantaroza	121.08 bcde	6.00 bcde	80.17 abcd	5.61 bcde
**20. **Karabola	111.42 def	5.33 cde	82.25 abcd	5.76 bcd
**21. **Kardia Vodiou	150.00 abc	7.53 ab	83.97 abcd	5.89 bcd
**22. **Takas	131.75 abcd	5.70 bcde	93.56 ab	5.72 bcd
**23. **Pastra	81.72 fg	4.97 de	64.17 e	5.36 cde
**24. **Milo Serron	97.84 efg	4.83 de	83.67 abcd	5.22 de
Average	124.40	6.40	80.59	5.66

* Varieties with the same letter within a column indicate no significant differences, according to the Duncan test (a = 0.05).

Regarding the number of inflorescences, which is indirectly related to the productive potential of the plants, the values ranged from 4.39 to 10.56 inflorescences *per* plant, as shown in **Table 3**. The cultivars “Aspros Lotos”, “Formula F1” (control), and “Milo Cephalonia” presented a higher number of inflorescences, with values of 8.42, 7.67, and 7.64 inflorescences per plant, respectively, though statistically significant differences were not observed. The “Imvros” cultivar had the lowest inflorescences, with an average of 4.25, as well as the “Boulgariki” cultivar, with 4.89.

In the same table (**Table 3**), the measurements corresponding to the height of tomato plants at the critical stage for appearance of the fourth inflorescence are shown. In this stage, most of the tomato cultivars had a height which ranged between 70 and 80 cm. The cultivars that had the lowest height, which is a desirable trait, were “Pastra” (64.17 cm), “Imvros” (74.50 cm), “Eratyras” (74.67 cm), and “Trikala Imathias” (75.33 cm). The cultivars “Takas” (93.56 cm) and “Milo Corfu” (92.34 cm) showed the fourth inflorescence at a higher height than the rest of the cultivars. In addition, the last column of **Table 3** shows the values of the length of internodes, a characteristic that did not differ significantly among the cultivars, noting values from 4.67 to 6.85 cm. The cultivars with the higher length of internode were “Milo Corfu”, “Atheras”, and “Filia Lesvou”, having statistically significant differences from the other cultivars. On the other hand, the “Imvros” had the lowest length of the internode (4.67 cm).

Regarding the size of leaf (**Table S2**) the cultivar “Imvros” showed the smallest leaf length, with 19.78 cm, while the cultivar “Feneou” had the largest, with 28.50 cm. Also, in the characteristic of leaf width, the values ranged from 14.72 cm (“Boulgariki”) to 22.81 cm (“Pastra”). As for the size of leaflets, the cultivars showed values from 7.33 cm (“Boulgariki”) to 11.53 cm (“Pastra”). In general, no major differences were found among the cultivars regarding the above characteristic. Based on the above characteristics (leaf length, leaf width, and size of leaflets) it is concluded that the cultivars “Evros”, “Pastra”, and “Feneou” have the largest leaves, while the “Boulgariki” and “Imvros” have the smallest.

The measurements of the morphological characteristics of the tomato fruit are presented in **Table 4**. As for the characteristics that indicate the size of the fruit (the polar diameter and equatorial diameter of the fruit), the cultivars “Takas”, “Imvrou”, “Evrou”, and “Nikoulas” stood out, having the largest polar diameter, with 69.78 mm, 65.51 mm, 65.10 mm, and 63.52 mm, respectively, while the largest equatorial diameter was recorded by the cultivar “Atheras”, which differed significantly from the other cultivars. In addition, the ratio of the two above characteristics indicates the shape of the fruit (Polar diameter/Equatorial diameter) and it showed statistically significant differences between the genetic materials (**Table 4**) used in this experiment. It was observed that the shape of the fruit ranged from flat to almost spherical shape. In particular, the cultivars approaching the value (1) have a spherical fruit shape, in contrast to the cultivars having values ​less or greater than 1 (UPOV-Tomato guidelines, 2011), which have a flat fruit shape.

**Table 4:** Average fruit morphological traits (size of peduncle scar, polar diameter, equatorial diameter, ratio length/diameter, thickness of pericarp, number of locules, and length peducle inside of fruit) according to the UPOV guideline (2011).

Fruit’s morphological characteristics							
Landraces	Polar diameter (mm)	Equatorial diameter (mm)	Ratio length/diameter	Size of peduncle scar (mm)	Thickness of pericarp (mm)	Number of locules	Length peducle inside of fruit (mm)
**1. **Filia Lesvou	49.54 cd*	56.12 bcd	0.88 defg	13.51 bc	4.84 bc	5.31 abcdef	10.21 bc
**2. **Atheras	59.99 abc	76.11 a	0.61 g	20.37 a	5.15 abc	7.33 abc	10.78 abc
**3. **Agion Oros	50.92 bcd	60.12 bc	0.84 efg	14.89 bc	6.43 a	5.05 bcdef	10.19 bc
**4. **Milo Chalkidiki	47.27 d	51.23 bcd	0.92 cdef	11.46 bcd	4.29 bc	4.03 f	12.33 abc
**5. **Souvritiki Evrou	52.27 bcd	46.60 d	1.14 abcd	10.41 cd	4.76 bc	4.00 f	6.59 bc
**6. **Boulgariki	45.66 d	53.76 bcd	0.85 efg	11.90 bcd	3.77 c	5.33 abcdef	4.92 c
**7. **Macedonia	49.61 cd	56.67 bcd	0.88 defg	14.45 bc	5.01 bc	4.50 cdef	8.26 bc
**8. **Milo Corfu	48.03 d	62.79 b	0.77 fg	15.96 ab	5.34 ab	6.00 abcdef	10.84 abc
**9. **Milo Cephalonia	40.88 d	54.03 bcd	0.76 fg	10.25 cd	4.67 bc	6.25 abcdef	8.02 bc
**10. **Imvros	65.51 a	55.61 bcd	1.18 abc	12.98 bc	4.65 bc	6.91 abcde	12.20 abc
**11. **Trikala Imathias	46.77 d	50.34 bcd	0.93 cdef	13.48 bc	4.82 bc	4.00 f	13.82 ab
**12. **Formula F1	48.83 cd	59.23 bcd	0.82 efg	12.17 bcd	5.31 ab	4.61 bcdef	11.50 abc
**13. **Eratiras	50.26 bcd	57.27 bcd	0.88 defg	13.66 bc	5.01 bc	5.42 abcdef	10.77 abc
**14. **Lotos	46.65 d	53.55 bcd	0.87 defg	10.66 bcd	4.72 bc	4.42 def	12.68 abc
**15. **Nikoulas	63.52 a	53.66 bcd	1.20 abc	12.93 bc	4.13 bc	6.89 abcde	10.41 bc
**16. **Evrou	65.10 a	48.76 cd	1.34 a	12.32 bcd	5.26 ab	4.23 ef	8.84 bc
**17. **Feneou	61.20 ab	56.63 bcd	1.08 abcde	12.17 bcd	4.48 bc	7.20 abcd	9.64 bc
**18. **Aspros Lotos	45.66 d	51.87 bcd	0.88 defg	10.36 cd	4.41 bc	4.94 bcdef	11.63 abc
**19. **Pantaroza	52.49 bcd	56.14 bcd	0.94 cdef	11.66 bcd	5.23 ab	8.00 a	12.81 abc
**20. **Karabola	48.58 d	52.31 bcd	0.94 cdef	12.65 bcd	4.76 bc	5.17 bcdef	9.21 bc
**21. **Kardia Vodiou	61.20 ab	58.31 bcd	1.06 bcde	7.49 d	5.54 ab	7.40 ab	18.61 a
**22. **Takas	69.78 a	55.67 bcd	1.25 ab	13.94 bc	4.80 bc	7.00 abcde	5.93 bc
**23. **Pastra	52.30 bcd	51.90 bcd	1.03 bcdef	12.14 bcd	5.46 ab	4.88 bcdef	8.39 bc
**24. **Milo Serron	47.02 d	53.66 bcd	0.88 defg	13.53 bc	4.70 bc	5.04 bcdef	8.95 bc
Average	52.40	55.65	0.94	12.70	4.91	5.47	10.30

* Varieties with the same letter within a column indicate no significant differences, according to the Duncan test (a = 0.05).

In the same table (**Table 4**), the values recorded by the cultivars regarding the characteristic “size of peduncle scar” are also presented. The smallest peduncle scar, which is desirable, was noticed in the cultivars “Kardia Vodiou” (7.49 mm), “Milo Cephalonia” (10.25 mm), and “Aspros lotos” (10.36 mm). On the other hand, the cultivars that had the largest peduncle scar size are “Atheras” (20.37 mm) and “Milo Corfu” (15.96 mm).

**Figure 2:**
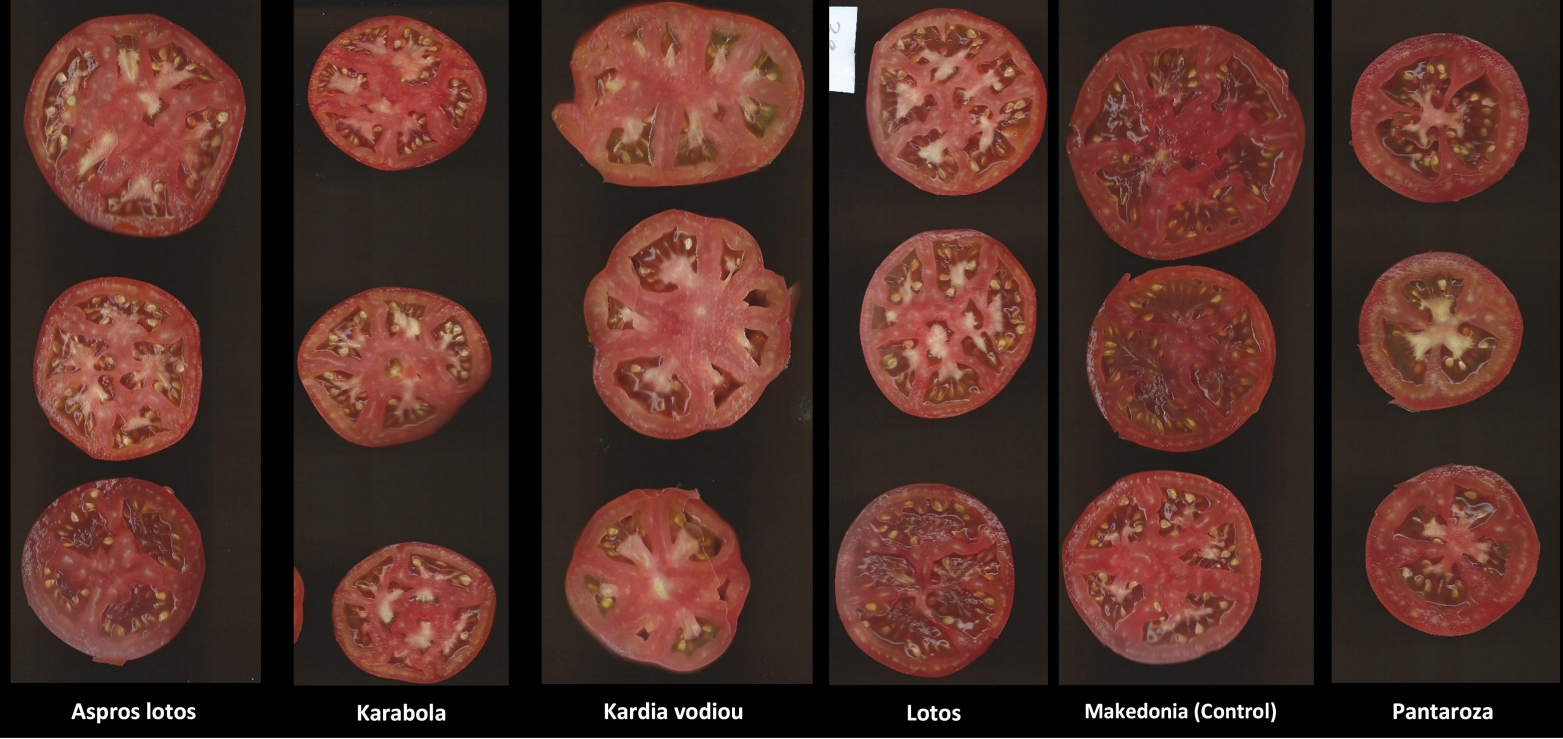
The vertical option of tomato fruits in five selected landraces and the control cv. “Macedonia”.

Regarding the thickness of the pericarp of the fruit (**Table 4**) – a characteristic associated with the post-harvest life of the fruit, as well as the texture in taste – the cultivar “Agion Oros” showed the highest value (6.43 mm), followed by the cultivars “Kardia vodiou” (5.54 mm) and “Pastra” (5.46 mm). The cultivars “Boulgariki” (3.77 mm) and “Nikoulas” (4.13 mm) also showed the lowest value of thickness of pericarp. As for the number of locules (**Table 4**), a characteristic that is positively correlated with the size and weight of the fruit, the values ranged from 4 to 8 locules. The lowest number of locules was observed in the cultivars “Souvritiki Evrou” and “Trikala Imathias”, while the highest number, in the cultivar “Pantaroza” (**Figure 2**). The cultivars “Kardia vodiou” and “Atheras” also showed a high number of locules, though statistically significant differences with “Pantaroza” were not observed. Finally, as for the undesirable feature “length of peduncle inside of the fruit” (**Table 4**), the values ​ranged from 4.92 mm to 18.61 mm. The cultivars “Kardia vodiou” and “Trikala Imathias” had the highest value, which is an undesirable trait, while “Boulgariki” and “Takas” had the lowest value for this characteristic.

### Yield components

Regarding the results of the early production, presented in **Table S3**, the “Formula F1” hybrid had the highest early yield production, with 1592.5 g. The following genotypes, “Aspros Lotos” with 1221 g, “Feneou” with 1197.15 g,” Pantaroza” with 1141.05 g and “Kardia Vodiou” with 1132.2 g, gave a high early yield, and statistically significant differences with the control F1 hybrid were not observed. The second control “Macedonia” had an early production of 928.63 g and thus eight cultivars showed superiority over it. As for the stability of performance (X/s), among the landraces used, the highest values were observed in “Kardia Vodiou”, “Nikolas”, “Pastra” and “Milo Serron.”

As for the number of fruits *per* plant of the early production (**Table S3**), “Aspros Lotos” was proved out the superior giving 12.77 fruits *per* plant, a value which is twice the number of the control “Macedonia” (**Table S3**). This landrace was the best, without having statistically significant differences with the “Formula F1” (hybrid), which gave 10.4 fruits per plant on average. It was then followed by the landraces “Milo Chalkidikis” with eight fruits, and “Lotos” with 7.67. The control “Macedonia” is in the seventh place among the cultivars evaluated (6.33 fruits per plant). The lowest value was observed in the cultivars “Milo Corfu” and “Imvros”, with an average of one and two fruits per plant, respectively. The landrace “Atheras” did not produce any fruit at the early harvest dates.

With regard to the average fruit weight at the early production (**Table S3**), the cultivars that stood out were “Milo Corfu”, “Imvros” and “Nikoulas”, having statistically significant differences with the control “Macedonia”, exhibiting an inbred vigor of 123%, 117%, and 93%, respectively. On the other hand, the lowest fruit weight was noticed in “Milo of Cephalonia” (59.77 g) and “Trikala Imathias” (94.53 g). In addition, a high value of stability of performance (X/s) was shown in the cultivars “Karabola”, “Formula F1” hybrid, and landrace “Pastra”.

The genotype’s averages for total production (yield/plant) are presented in **Table 5** and **Figure S1**. As compared to the “Formula F1” hybrid used as control, the landrace “Feneou” produced more, giving a total yield of 2471.5 g, but statistically significant differences with hybrid (Formula F1) were not observed. “Formula F1” followed with 2311.17 g of total production, while the second control “Macedonia” was fifth in the ranking of 24 cultivars, falling behind the first cultivar (“Feneou”) by 54%. The third best performance was recorded by “Pantaroza”, with 2084.95 g. All the above cultivars did not differ significantly from each other statistically. The cultivars “Milo Corfu”, “Takas”, and “Souvritiki Evrou” were classified into the final of the list for total production. In terms of stability of performance (X/s), the genetic materials that showed the highest values were the “Eratyras” cultivar (4.4) and the “Feneou” cultivar (4.0), which had the biggest total yield. The genetic materials with the lowest stability of performance were “Filia Lesvou”, “Souvritiki Evrou”, and “Pastra” with a value of 4.0.

**Table 5:** Total fruit yield (yield/plant, number of fruits/plant, and weight/fruit), vigor/depression (% of pure line “Macedonia”), and stability of performance (x/s) of the landraces.

	Total Fruit yield								
	Yield/plant (g)			Number of fruits/plant			Weight/fruit (g)		
	x	V/D	x/s	x	V/D	x/s	x	V/D	x/s
1. Filia Lesvou	1135.30 cdefg*	71	1.2	5.27 ghijk	42	2.2	161.30 bcdef	128	2.0
2. Atheras	1273.80 bcdefg	79	2.5	4.83 hijk	39	2.3	261.93 a	207	3.2
3. Agion Oros	1326.03 bcdefg	83	1.5	8.77 cdefghijk	70	1.7	142.10 bcdef	112	1.5
4. Milo Chalkidiki	1460.27 abcdefg	91	1.9	15.00 abc	120	1.6	102.00 def	81	5.1
5. Souvritiki Evrou	656.87 fg	41	1.2	4.33 ijk	35	1.2	149.53 bcdef	118	3.3
6. Boulgariki	774.47 fg	48	1.6	5.90 fghijk	47	2.3	124.60 bcdef	98	2.5
7. Macedonia	1603.53 abcdef	-	2.3	12.50 bcdef	-	2.0	126.50 bcdef	-	3.4
8. Milo Corfu	485.63 g	30	3.4	2.33 k	19	3.3	213.83 ab	169	3.1
9. Milo Cephalonia	806.40 efg	50	1.3	11.60 bcdefgh	93	1.3	71.93 f	57	2.6
10. Imvros	1030.40 defg	64	2.2	4.00 ijk	32	2.0	254.58 a	201	3.4
11. Trikala Imathias	711.90 fg	44	2.5	9.83 cdefghij	79	3.3	71.47 f	56	5.0
12. Formula F1	2311.17 ab	144	1.7	17.40 ab	139	1.7	129.43 bcdef	102	7.7
13. Eratiras	1568.07 abcdef	98	4.4	10.67 bcdefghi	85	3.6	151.53 bcdef	120	4.4
14. Lotos	1566.13 abcdef	98	2.7	13.00 bcde	104	2.5	124.00 bcdef	98	5.3
15. Nikoulas	1843.47 abcde	11	2.3	7.77 defghijk	62	1.4	251.00 a	198	1.4
16. Evrou	1071.63 bcdefg	67	2.5	8.20 cdefghijk	66	2.3	139.83 bcdef	111	2.5
17. Feneou	2471.50 a	154	4.0	13.75 abcd	110	3.1	187.50 abcd	148	5.2
18. Aspros Lotos	1885.83 abcd	118	2.4	19.67 a	157	2.2	95.50 ef	75	4.5
19. Pantaroza	2084.95 abc	130	2.4	12.10 bcdefg	97	1.5	179.70 abcde	142	3.4
20. Karabola	880.30 defg	55	1.6	6.17 efghijk	49	1.6	141.53 bcdef	112	4.4
21. Kardia Vodiou	1481.77 abcdefg	92	2.2	7.00 defghijk	56	2.1	205.07 abc	162	3.7
22. Takas	598.37 fg	37	1.4	3.00 jk	24	1.8	214.70 ab	170	1.7
23. Pastra	964.53 defg	60	1.2	7.33 defghijk	59	1.9	120.97 cdef	96	3.1
24. Milo Serron	1366.20 bcdefg	85	2.5	9.85 cdefghij	79	2.5	136.40 bcdef	108	5.4
Average	1306.60	**-**	**-**	9.18	**-**	**-**	155.85	-	-

* Varieties with the same letter within a column indicate no significant differences, according to the Duncan test (a = 0.05).

Regarding the number of fruits per plant of the total production (**Table 5**), the “Aspros Lotos” had a higher number (19.67 fruits/plant) and showed 57% superiority over control “Macedonia” (12.5 fruits/plant). This was followed by the hybrid “Formula F1” (17.4 fruits/plant) and the “Milo Chalkidiki” (15 fruits/plant). The cultivars with the smallest number of fruits were “Milo Corfu”, “Takas”, and “Imvros”. The highest value for stability of performance (X/s) was observed in “Eratyras” cultivar, with a value of 3.6.

The cultivars with the highest average fruit weight “Atheras”, “Imvros”, and “Nikoulas” – with a value of over 250 g per fruit – differentiated significantly from both controls (**Table 5**). The lowest average fruit weight was observed in the cultivars “Trikala Imathias”, “Milo Cephalonia”, and “Aspros lotos”. The stability of performance (X/s) generally presented high values. Among them, the “Formula F1” hybrid had the highest, with 7.7, as well as the “Milo Serron”, with 5.4. The cultivars “Nikoulas”, with 1.4, and “Agion Oros”, with 1.5, showed the lowest stability of performance (X/s) among all the landraces.

### Fruit quality and nutritional value traits

The pH analysis showed statistically significant differences and ranged from 3.71 to 4.82. As presented in **Table 6**, the “Feneou” cultivar showed the highest value (4.82). High pH values were also recorded in the cultivars “Milo Serron” and “Karabola”, with a value of 4.52. On the other hand, the cultivars “Milo Cephalonia” and “Imvros” had the lowest pH, with values of 3.71 and 3.76, respectively. The juice of control Macedonia showed a mean value of pH close to 3.89.

**Table 6:** Average pH, acidity (% citric acid), and total soluble solids (°Brix), and vigor/depression (% of pure line “Macedonia”), for the landraces.

Landrace	Quality traits					
	pH		Acidity(% citric acid)		Total soluble solids(°Brix)	
	x	V/D	x	V/D	x	V/D
1. Filia Lesvou	3.92 m*	101	3.94 c	152	4.43 h	102
2. Atheras	4.32 e	111	2.88 m	111	4.87 e	111
3. Agion Oros	4.18 h	108	2.98 k	115	4.17 k	95
4. Milo Chalkidiki	4.29 f	110	2.85 m	110	5.37 c	123
5. Souvritiki Evrou	4.29 f	110	3.86 d	149	4.27 j	98
6. Boulgariki	4.43 c	114	3.15 h	122	5.47 b	125
7. Macedonia	3.89 o	-	2.59 p	-	4.37 hi	-
8. Milo Corfu	4.10 j	105	2.92 l	113	4.07 h	93
9. Milo Cephalonia	3.71 s	95	3.35 f	130	3.67 n	84
10. Imvros	3.76 r	97	2.68 o	103	3.80 m	87
11. Trikala Imathias	3.86 p	99	2.57 p	99	4.87 e	111
12. Formula F1	4.22 g	109	4.07 a	157	4.57 fg	105
13. Eratiras	3.84 q	99	3.86 d	149	4.33 ij	99
14. Lotos	4.14 i	106	2.91 l	113	4.43 h	102
15. Nikoulas	3.90 n	100	3.76 e	145	4.17 k	95
16. Evrou	4.00 l	103	4.02 b	156	4.53 g	104
17. Feneou	4.82 a	124	3.00 k	116	5.03 d	115
18. Aspros Lotos	4.52 b	116	3.21 g	124	5.57 a	127
19. Pantaroza	4.30 f	110	3.03 j	117	4.63 f	106
20. Karabola	4.52 b	116	2.78 n	107	4.83 e	111
21. Kardia Vodiou	4.06 k	104	3.92 c	151	4.37 hi	100
22. Takas	4.22 g	109	4.07 a	157	4.57 fg	105
23. Pastra	4.35 d	112	3.09 i	119	5.37 c	123
24. Milo Serron	4.52 b	116	3.21 g	124	5.57 a	127
Average	4.17	–	3.94 c	–	4.64	–

* Varieties with the same letter within a column indicate no significant differences, according to the Duncan test (a = 0.05).

The average citric acid content (%) (**Table 6**), which determines the acidity of the fruit and together with the total soluble solids is mainly responsible for the taste of fruits. The cultivars showed statistically significant differences and the values ​ranged from 2.57 (cultivar “Trikala Imathias”) to 4.07 (“Formula F1” and “Takas”). All cultivars, except the cultivar “Trikala Imathias”, had higher acidity value than the control “Macedonia”.

With regard to the total soluble solids (**Table 6**), a characteristic which is very important for the taste and sensory properties of the fruit, the cultivars had statistically significant differences. The cultivars with the highest content were “Aspros Lotos” and “Milo Serron”, with a value of 5.57, having a significant superiority from both controls. This was followed by the “Bulgariki” cultivar (5.47), and “Pastra” cultivar (5.37). The landraces that showed the greatest lag compared to the control Macedonia were “Milo Cephalonia” and “Imvros”, with lag rates of 16% and 13%, respectively.

The nutritional characteristics of the fruit include ascorbic acid (mg/100 g), lycopene (mg/100 g), total carotenoids mg/100 g), and total phenolics (mg/100 g). Regarding the ascorbic acid (**Table 7**), the cultivars “Aspros Lotos” and “Milo Serron” were superior to the control Macedonia by 31%, with 32.11 mg/100 g of ascorbic acid. The cultivars “Bulgariki”, “Milo Chalkidiki”, and “Pastra” followed. The cultivars “Milo Corfu”, “Milo Chefalonia”, and “Imvros” had almost the half of ascorbic acid content which is less than 18 mg/100 g.

**Table 7:** Average ascorbic acid (mg/100 g), lycopene (mg/100 g), carotenoids (mg/100 g) and phenols (mg/100 g), and vigor/depression (% of pure line “Macedonia”), for the landraces.

Landrace	Nutritional value traits							
	Ascorbic acid (mg/100 g)		Lycopene(mg/100 g)		Carotenoids(mg/100 g)		Phenols(mg/100 g)	
	x	V/D	x	V/D	x	V/D	x	V/D
1. Filia Lesvou	21.57 l*	88	13.09 hi	90	17.40 de	91	27.28 cdef	111
2. Atheras	27.30 f	111	16.18 b	111	20.79 ab	108	27.34 cdef	111
3. Agion Oros	18.28 o	74	11.59 k	80	15.71 fgh	82	21.68 h	88
4. Milo Chalkidiki	31.39 b	128	11.26 l	77	14.35 hi	75	28.57 cd	116
5. Souvritiki Evrou	20.58 n	84	12.45 j	86	16.54 efg	86	25.35 fg	103
6. Boulgariki	31.50 b	128	15.84 c	109	20.47 ab	107	31.72 a	129
7. Macedonia	24.57 i	-	14.54 e	-	19.20 bc	-	24.61 g	-
8. Milo Corfu	17.91 p	73	11.36 kl	78	15.59 gh	81	21.25 h	86
9. Milo Cephalonia	17.81 p	72	10.77 m	74	14.42 hi	75	21.94 h	89
10. Imvros	16.49 q	67	10.30 n	71	13.27 i	69	19.71 h	80
11. Trikala Imathias	28.26 e	115	10.12 n	70	12.82 i	67	25.72 efg	105
12. Formula F1	22.86 j	93	14.71 e	101	19.65 bc	102	28.21 cde	115
13. Eratiras	21.14 m	86	12.83 i	88	15.77 fgh	82	26.74 defg	109
14. Lotos	25.83 h	105	15.28 d	105	20.34 ab	106	25.78 efg	105
15. Nikoulas	21.14 m	86	13.59 g	93	18.15 cde	95	26.08 defg	106
16. Evrou	22.01 k	90	13.35 gh	92	17.26 def	90	27.83 cde	113
17. Feneou	25.76 h	105	15.50 d	107	20.74 ab	108	27.92 cde	113
18. Aspros Lotos	32.11 a	131	16.14 b	111	20.72 ab	108	32.33 a	131
19. Pantaroza	26.88 g	109	15.91 bc	109	20.93 ab	109	26.83 cdefg	109
20. Karabola	29.15 d	119	16.89 a	116	22.08 a	115	29.24 bc	119
21. Kardia Vodiou	22.00 k	90	14.16 f	97	18.62 cd	97	27.15 cdef	110
22. Takas	22.86 j	93	14.71 e	101	18.49 cd	96	28.21 cde	115
23. Pastra	30.89 c	126	15.52 d	107	20.48 ab	107	31.11 ab	126
24. Milo Serron	32.11 a	131	16.13 b	111	20.71 ab	108	32.33 a	131
Average	24.60	–	13.84	–	18.10		26.87	–

* Varieties with the same letter within a column indicate no significant differences, according to the Duncan test (a = 0.05).

In **Table 7** and in **Figure S2**, the fruit lycopene, the most important antioxidant in tomato, ranged from 10.12 to 16.89 (mg/100 g). The highest value was observed in the cultivar “Karabola” (16.89 mg/100 g), showing significant differences from all the cultivars statistically, followed by the cultivars “Atheras” (16.18 mg/100 g), “Aspros Lotos” (16.14), and “Milo Serron” (16.13 mg/100 g). The cultivar “Trikala Imathias” (10.12 mg/100 g) showed the lowest lycopene content. Also, the cultivars “Milo Chefalonia” and “Imvros” are classified again at the last positions of the list for lycopene content (**Table 7**), with significant percentages of lag from the control.

The analyses for the average carotenoid content (mg/100 g) indicated that “Karabola” landrace showed the highest value (22.08 mg/100 g), differing significantly from the control “Macedonia” statistically (**Table 7**). The cultivars “Pantaroza” (20.93 mg/100 g), “Atheras” (20.79 mg/100), “Feneou” (20.74 mg/100), “Aspros Lotos” (20.72 mg/100), and “Milo Serron” (20.71 mg/100 g) also recorded higher carotenoid content. The control “Macedonia” was in the 11^th^ place of the ranking, with a value of 19.20 mg/100 g. The cultivars “Imvros” (13.27 mg/100 g) and “Trikala Imathias” (12.82 mg/100 g) were ranked lower in the order (**Table 7**).

The same table (**Table 7**) presents the results of the analyses for the average total phenolic content (mg/100g). The cultivars “Aspros Lotos” and “Milo Serron” recorded the highest value, with an average content of 32.33 mg/100 g. This was followed by the cultivars “Boulgariki” (31.72 mg/100g), “Pastra” (31.11 mg/100g), and “Karabola” (29.24 mg/100g). The lowest value of total phenolics was shown by the cultivars “Milo Corfu” and “Imvros”, cultivars that showed low values in all analyses regarding the nutritional value of the fruit.

**Figure 3:**
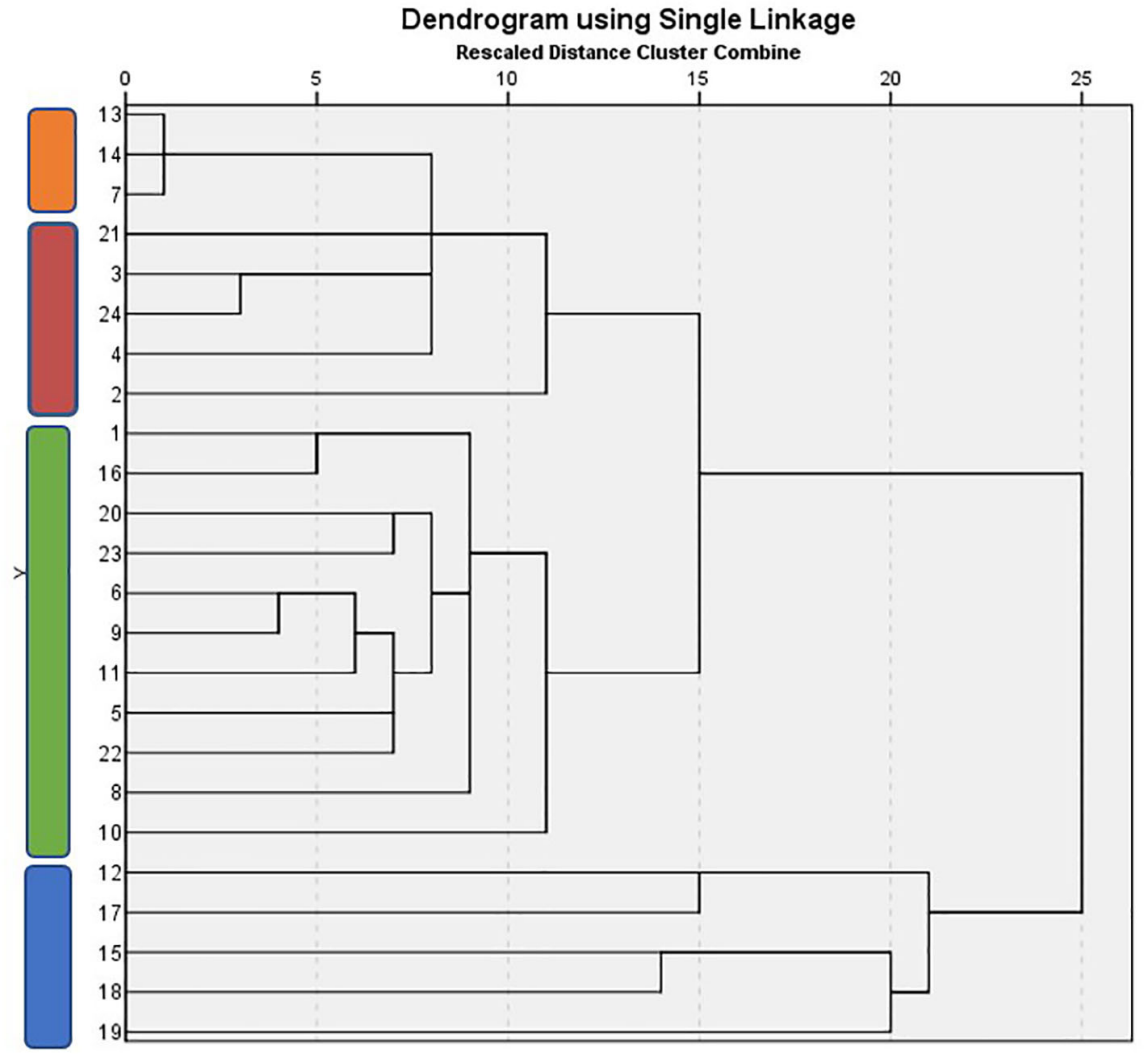
Relationships among tomato landraces based on five main attributes used as criteria for the pure line selection method.

Hierarchical cluster analysis was carried out with the aid of Pearson coefficients, resulting in a dendrogram construction for yield and fruit quality attributes (**Figure 3**). The tomato landraces tested in this study were clearly grouped in three main groups. The first group is subdivided into two subgroups including the landraces “Eratiras”, “Lotos”, “Macedonia” (first subgroup), “Kardia Vodiou”, “Agion Oros”, “Milo Serron”, “Milo Chalkidiki” and “Atheras” (second subgroup). The second group includes the cultivars “Filia Lesvou”, “Evrou”, “Karabola”, “Pastra”, “Boulgariki”, “Milo Cephalonia”, “Trikala Imathias”, “Souvritiki Evrou”, “Takas”, “Milo Corfu”, and “Imvros”. The third group consists of the cultivars “Formula F1”, “Feneou”, “Nikoulas”, “Aspros lotos” and “Pantaroza”.

Most of the selected landraces (13, 14, 21, 4) for the second phase belong to the first group; two of them originate from the third group (18, 19), and only one (20) originates from the second group. These landraces originate from different geographical areas and their only common characteristics are the growth type and shape of fruits. Landraces were clearly distinguished from commercial cultivars (cv. “Macedonia” and “Formula F1”), which belong to the second group. Based on the second and third cluster group, it can be concluded that among the 22 landraces, four from seven selected landraces are well separated and distinct from each other.

### Phase 2 – Yield components

After applying the pure line selection method for one year, the seven selected cultivars were evaluated for yield components (**Table 8**). Specifically, in terms of total yield, the cultivar “Pantaroza” stood out, with an average total yield of 4195.63 g, significantly differing from the control “Macedonia” and showing superiority of 92%. The cultivars “Aspros Lotos” and “Kardia Vodiou” followed with a yield of 3493.20 g and 3492.02 g, respectively. These landraces perform better than the “Formula F1” hybrid regarding the yield in the low-input environment of evaluation.

**Table 8:** Total fruit yield (yield/plant, number of fruits/plant, and weight/fruit), vigor/depression (% of pure line “Macedonia”), and stability of performance (x/s) of the seven selected landraces.

Landrace	Total fruit yield								
	Yield/plant (g)			Number of fruits/plant			Weight/fruit (g)		
	x	V/D	x/s	x	V/D	x/s	x	V/D	x/s
1. Milo Chalkidiki	2390.11 b*	110	2.5	15.97 b	115	2.5	151.79 b	93	7.5
2. Macedonia	2181.94 b	-	3.0	13.87 b	-	2.9	162.52 b	-	5.2
3. Formula F1	2992.50 ab	137	3.0	19.26 b	139	3.2	159.58 b	98	6.3
4. Eratiras	2941.13 ab	135	3.0	18.40 b	133	3.0	159.82 b	98	6.6
5. Lotos	2535.10 b	116	1.6	18.42 b	133	2.0	131.93 b	81	3.8
6. Aspros Lotos	3493.20 ab	160	3.1	28.40 a	205	3.8	123.03 b	76	7.3
7. Pantaroza	4195.63 a	192	2.9	29.07 a	210	3.1	144.70 b	89	3.6
8. Karabola	2882.19 ab	132	2.4	17.23 b	124	2.5	163.58 b	101	4.4
9. Kardia Vodiou	3492.02 ab	160	2.3	14.40 b	104	3.5	238.21 a	147	3.6
Average	3011.54	-	-	19.45	–	–	159.46	-	-

The three yield components used as criteria of selection.

* Varieties with the same letter within a column indicate no significant differences, according to the Duncan test (a = 0.05).

The number of fruits per plant (**Table 8**) was higher in all cultivars in comparison to the control cv. “Macedonia”. Specifically, in Phase 2, some of the landraces, like “Aspros Lotos” and “Pantaroza” showed vigor, having a number of fruits higher by 105% and 110% compared to the cv. “Macedonia”. The highest performance was recorded by the cultivars “Pantaroza” and “Aspros Lotos”, which stood out significantly from the rest of the cultivars statistically, with values of 29.07 and 28.40 fruits per plant, respectively. The cultivar “Kardia Vodiou” had the lowest number of fruits, but at the same time it recorded the highest fruit weight, with 238.21 g, and was distinguished from the rest of the cultivars (**Table 8**). The cultivar “Karabola” had the second largest fruit weight, followed by the control “Macedonia” with small difference (**Table 8**).

### Phase 2 – Nutritional value traits

In the seven selected cultivars, as well as in the controls “Macedonia” and “Formula F1”, an analysis of the content of lycopene and carotenoids in the fruit was carried out, which is presented in **Table 9**. The concentration of lycopene in the selected landraces ranged from 12.4 to 20.14 mg/100g. The cultivar “Kardia vodiou” had the highest lycopene content, with an extremely high value of 20.14 mg/100g, followed by the cultivar “Aspros lotos”, with 18.21 mg/100g. The cultivars “Milo Chalkidiki” and “Eratiras” had the lowest lycopene content, with values of 12.41 and 13.01 mg/100g, respectively. The value of the control cv. Macedonia was close to the average of all cultivars used in this experiment (**Table 9**).

The cultivars “Aspros Lotos” (23.54 mg/100g), and “Pantaroza” (21.88 mg/100g) stood out in terms of carotenoid content (**Table 9**). All the selected cultivars managed to surpass the control cv. “Macedonia”, except the cultivar “Milo Chalkidiki”, which again lagged the control by 11%.

**Table 9:** Average lycopene (mg/100 g) and carotenoids (mg/100 g) and vigor/depression (% of pure line “Macedonia”), for the seven selected landraces.

Landrace	Nutritional value traits			
	Lycopene(mg/100 g)		Carotenoids(mg/100 g)	
	x	V/D	x	V/D
1. Milo Chalkidiki	12.41 f*	82	15.03 g	89
2. Macedonia	15.06 d	-	16.83 h	-
3. Formula F1	15.40 d	102	18.44 e	110
4. Eratiras	13.01 e	86	16.90 f	100
5. Lotos	15.83 d	105	18.55 d	110
6. Aspros Lotos	18.21 b	121	23.54 a	140
7. Pantaroza	16.33 c	108	21.88 b	130
8. Karabola	15.76 d	105	20.83 c	124
9. Kardia Vodiou	20.14 a	134	21.03 b	125
Average	15.79	–	18.69	–

These two nutritional value characteristics used as criteria of selection.

* Varieties with the same letter within a column indicate no significant differences, according to the Duncan test (a = 0.05).

## Discussion

This study provides an extensive evaluation of the genetic diversity in a collection of 22 most important and remunerative Greek tomato landraces for “fresh market” and two commercial cultivars (Macedonia/Pure Line and Formula F1 Hybrid) which are currently used in the Greek tomato cultivation areas. The role of landraces and indigenous plant species is gaining increasing attention because of: (a) changing climatic conditions all over the world, (b) the necessity to produce more environmentally friendly food products, and (c) the demand of consumers for healthier and safer food.

### Useful genetic variability

Tomato local cultivars are genetic populations of an autogamous species having high variability related to adaptation, resistance to abiotic conditions, or adaptation to low inputs and acceptable sensory properties (Figàs et al., 2018). For these reasons, landraces are ideal genetic material (gene pool) for exploitation, directly or after the application of mild breeding approaches focused on all the above new human demands.

**Figure 4:**
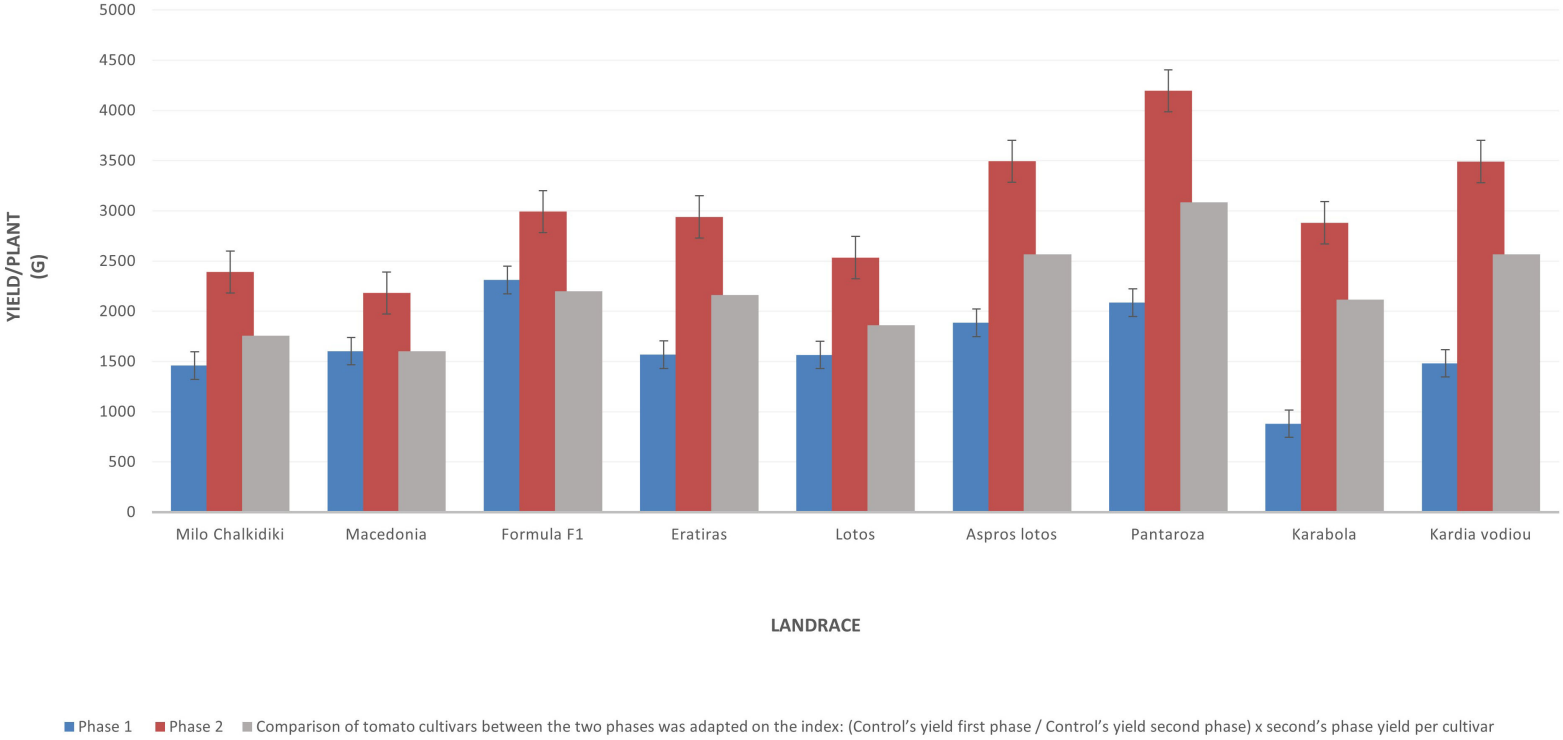
A comparative evaluation between the first phase and second phase after the selection for YIELD in seven selected tomato landraces and the two controls (Macedonia and Formula F1).

In our study, we realized the dynamic of tomato landraces, especially when cultivated under low-input organic conditions and the high probability to select specific genotypes in order to improve their yield and nutritional properties. The screening of this collection of tomato landraces from Greece gave us the opportunity to reveal the existence of high genetic variability in many important characteristics, including different fruit types and shapes, yield potential parameters, as well as fruit quality and nutritional traits. Landraces like “Imvros” are characterized by low stature and others, like “Kardia Vodiou”, “Pantarosa”, and “Karabola”, have an interesting shape of fruits (**Table 4** and **Figure 2**). The yield potential of starting populations in landraces “Aspros Lotos”, “Lotos”, “Milo Chalkidikis”, “Feneou”, “Pantaroza”, and “Kardia Vodiou” is particularly high; even in the first year of evaluation, the growing conditions were not the most favorable. Generally, the landraces have lower yield potential compared to the commercial cultivars, although in the present study the yield of the above landraces did not present statistically significant differences from the controls used. This observation is in agreement with Mazzucato et al. (2008) and Corrado et al. (2014). It is important to notice that the “F1 Formula” hybrid gave a satisfactory yield, which is better than the local cultivars, even though it was grown under low-input conditions. This view is reversed later, after 2 years of selection under low-input organic conditions, where landraces “Pantarosa”, “Aspros Lotos”, “Eratyras”, and “Kardia Vodiou” were equal or surpassed the total yield of commercial cultivars “Macedonia” and the “F1 Formula” hybrid (**Figure 4**).

**Figure 5:**
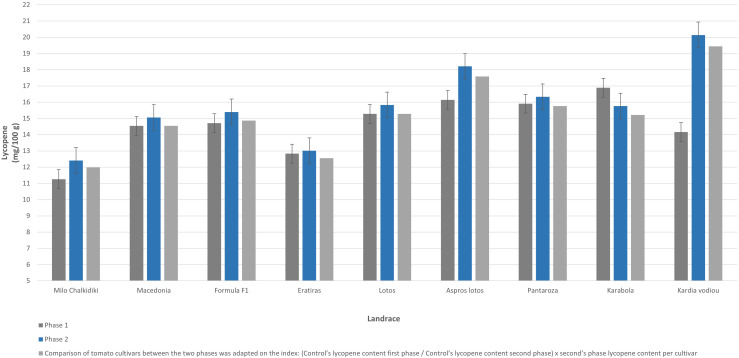
The comparison of the content of lycopene in tomato fruits (mg/100g) of the seven selected landraces and the two controls for the two seasons.

**Figure 6:**
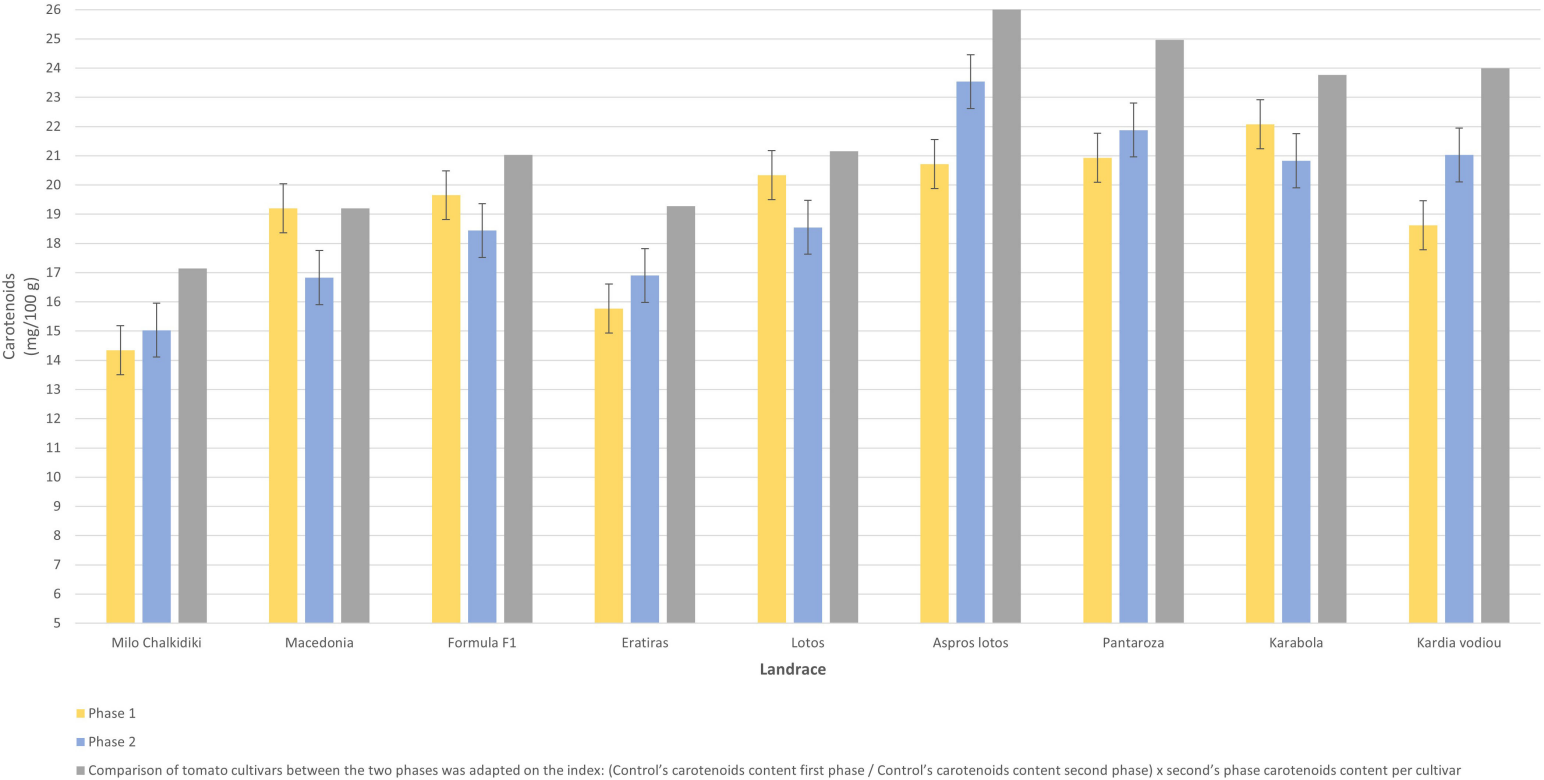
The comparison of the content of carotenoids in tomato fruits (mg/100g) of the seven selected landraces and the two controls for the two seasons.

The differences in bioactive compound concentration among tested tomato landraces under the same environment of evaluation is due to the genetic background of these cultivars. The lycopene content in fruits ranged from 12 to 20 mg/100g. In landraces like “Aspros Lotos”, “Pantarosa”, and “Karabola”, characterized by high concentration in lycopene (**Figure 5**), the percentage ranged from 16.3 to 20.4, which is equal to or higher than standards referred to literature (Figàs et al., 2015). Furthermore, the fruits of these landraces could be characterized as functional food, because of their high concentration in total carotenoids (**Figure 6**). Especially, the landrace “Aspros Lotos” gave a higher concentration in total carotenoids, one of the highest values referred in the literature (Gonzalez-Cebrino et al., 2011) for organic culture.

### Effect of the farming system

The results obtained show that the organic farming system contributed significantly to the accumulation of bioactive compounds like lycopene and carotenoids. These results agree with the findings of Oliveira et al. (2013), who reported that organic tomatoes accumulate a high concentration of bioactive compounds because of increased stress (low-input) conditions, to which plants are exposed in organic farming. Furthermore, Vinha et al. (2014) showed through their experiments that the use of the agricultural method and farming system influences not only the contents but also the distribution of bioactive compounds in the fruit. This research group indicated that organic tomatoes contained higher levels of lycopene, vitamin C, carotenoids, and phenolic compounds. Hallmann (2012) noticed that the farming system and dates for maturation of tomato fruits influence the concentration of soluble sugars. According to her experimental data, the organic farming system contributed significantly to the accumulation of total sugars in tomato fruits. Positive correlations were found between the antioxidant activity and the contents of bioactive compounds, especially for total phenolics and flavonoids. Vinha et al. (2014) reached the conclusion that in a nutritional perspective, the organic tomatoes analyzed were healthier than those produced by conventional practices.

### Breeding method and opportunities

The previous studies (Mavromatis et al., 2013) proved that pure line selection (PLS) is a promising and mild method to improve tomato landraces effectively for yield components. Into this study, following a combined selection and using two qualitative (lycopene and total carotenoids content in tomato fruits) and three quantitative (number of fruits per plant, weight of fruits per plant and total commercial yield) parameters as criteria, we succeed to increase the yield potential up to 110% by mean and sometimes up to twice as in case of “Kardia Vodiou” and “Pantaroza”. Many researchers support that high yield potential is related to the number of fruits per plant and fruit weight (Avdikos et al., 2022a). Given their high path coefficient value and significant correlation (p< 0.05) with the yield potential, these two characteristics are highly recommended to be used as selection criteria for high yield potential of tomato plants. Furthermore, a significant improvement in major yield component (number of fruits per plant) for the most landraces used, at a level up to 105% higher than the control (“Macedonia”) and sometimes up to twice in comparison to the initial population of each landrace, confirms the reliability of the PLS breeding method.

The application of the PLS method succeeds to increase the level of lycopene up to units (mg/100 g) in all the landraces used in this experiment, except for “Karabola”. Although many factors (growing, climatic and soil conditions, farming system and year of experimentation) influence the concentration of bioactive compounds into tomato fruits (Hallmann, 2012), into our experiments we followed selection through PLS and succeeded in increasing the concentration in total carotenoids and lycopene in almost all landraces.

## Conclusions

An integrated description of traditional tomato cultivars, including the morphological, physicochemical, and nutritional parameters was attempted. This study envisaged the characterization and further exploitation of tomato landraces, especially when cultivated under organic or low-input conditions. Using a combined multivariate analysis based on the three main quantitative yield components and two qualitative nutritional parameters related to nutritional value, we succeeded to identify the most promising landraces, which afterwards effectively improved (cv. “Pantaroza”, cv. “Kardia Vodiou” and cv. “Aspros Lotos”), following a short period (2 years) of a breeding scheme based on the PLS method, under low-input farming conditions. This approach provides information for the nutritive value and specific utilization of tomato landraces. All these data are expected to be used for direct exploitation or indirect participation into tomato breeding, supporting the interest of organic farmers and food processors, for the production of high nutritive tomato end products, with a low carbon footprint for the environment.

## Data availability statement

The raw data supporting the conclusions of this article will be made available by the authors, without undue reservation.

## Author contributions

AM, RT, and IA wrote the manuscript. AM and IA designed the experiments. RT, AG, and IN applied analysis on physicochemical and nutritional traits RT, IA, KK, carried field experimentation and AM, RT, IA wrote the R scripts for the statistical analyses. All authors contributed to the article and approved the submitted version.

## Supplementary material

Supplementary Figure 1 The total production (yield per plant) of 22 landraces and two tomato cultivars used as controls. Commercial cultivars presented on the bold columns. The dotted line shows the average yield per plant.

Supplementary Figure 2 The content of lycopene in tomato fruits (mg/100g) of 22 landraces and two tomato commercial cultivar used as controls.
