# Supplementary figures and images for: Characterization and evaluation of Greek tomato landraces for productivity and fruit quality traits related to sustainable low-input farming systems
Source: Front Plant Sci. 2022 Dec 12;13:994530. doi: 10.3389/fpls.2022.994530 (PMC9791058; doi:10.3389/fpls.2022.994530)

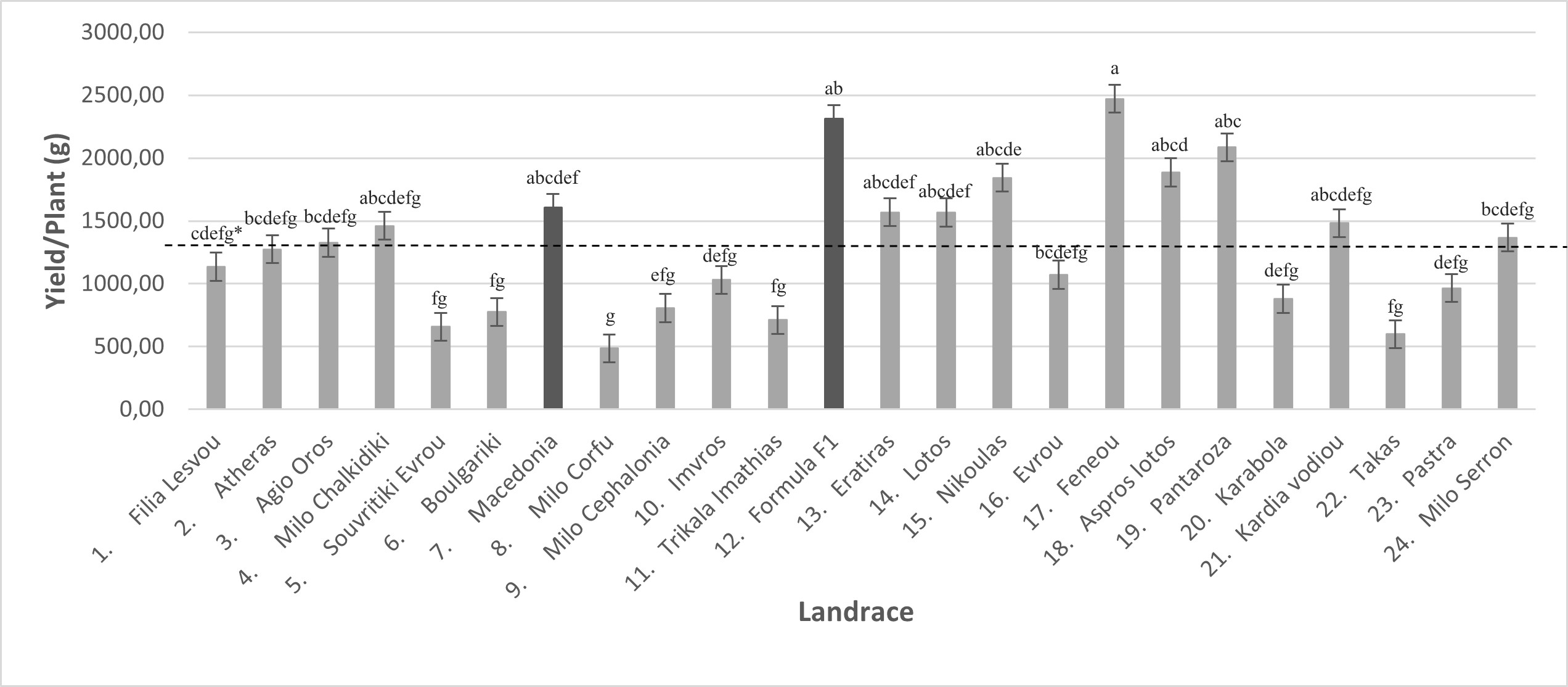

Supplement: Supplementary Figure 1 — The total production (yield per plant) of 22 landraces and two tomato cultivars used as controls. Commercial cultivars presented on the bold columns. The dotted line shows the average yield per plant. [file Image_1.jpeg]

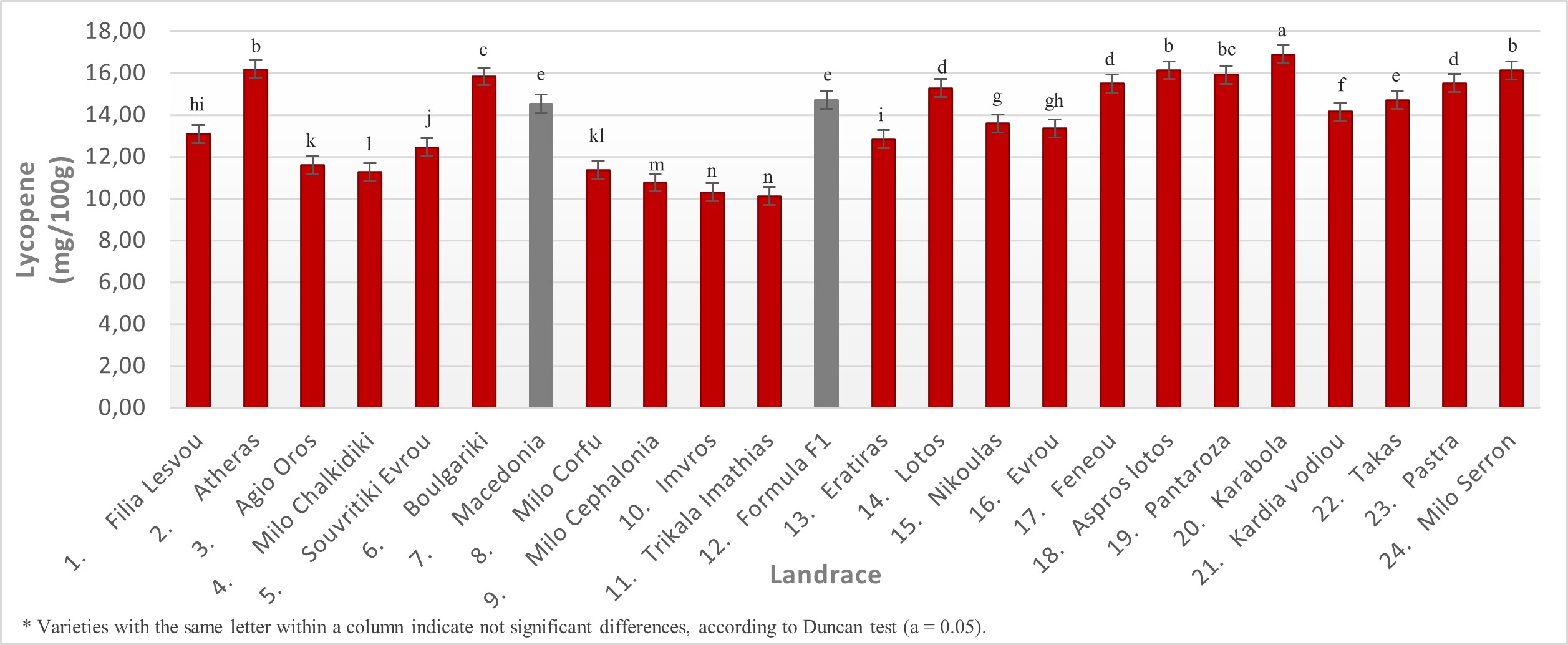

Supplement: Supplementary Figure 2 — The content of lycopene in tomato fruits (mg/100g) of 22 landraces and two tomato commercial cultivar used as controls. [file Image_2.jpeg]
