# Supplementary material for: Characterization and evaluation of Greek tomato landraces for productivity and fruit quality traits related to sustainable low-input farming systems
Source: Front Plant Sci. 2022 Dec 12;13:994530. doi: 10.3389/fpls.2022.994530 (PMC9791058; doi:10.3389/fpls.2022.994530)
Supplement: Supplementary file 3 [file Table_1.docx]

**Table S1│** Phenotypical (morphological) characteristics, measured according to UPOV guideline

| Α/Α | morphological characteristics |
| --- | --- |
| 1 | **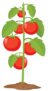Plant:** height (cm) |
| 2 | **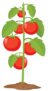Plant:** height of 4th inflorescence (cm) |
| 3 | **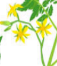 Inflorescence:** number of inflorescences |
| 4 | **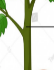Stem:** length of internode (cm) |
| 5 | **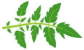Leaf:** length (cm) |
| 6 | **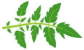Leaf:** width (cm) |
| 7 | **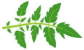Leaf:** size of leaflets (cm) |
| 8 | **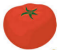Fruit:** size of peduncle scar (mm) |
| 9 | **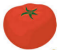Fruit:** polar diameter (mm) |
| 10 | **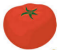Fruit:** equatorial diameter (mm) |
| 11 | **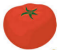Fruit:** ratio length/diameter |
| 12 | **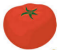Fruit:** thickness of pericarp (mm) |
| 13 | **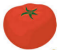Fruit:** number of locules |
| 14 | **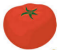Fruit:** length peducle inside of fruit (mm) |
