# Supplementary material for: Characterization and evaluation of Greek tomato landraces for productivity and fruit quality traits related to sustainable low-input farming systems
Source: Front Plant Sci. 2022 Dec 12;13:994530. doi: 10.3389/fpls.2022.994530 (PMC9791058; doi:10.3389/fpls.2022.994530)
Supplement: Supplementary file 4 [file Table_2.docx]

**Table S2 │** Average leaf’s morphological traits (length of leaf, width of leaf and size of leaflets) according to Upov guideline (2011).

| Leaf’s Morphological Characteristics | | | |
| --- | --- | --- | --- |
| Landraces | **Leaf: length (cm)** | **Leaf: width (cm)** | **Leaf: size of leaflets (cm)** |
| 1. Filia Lesvou | 23.75 def* | 17.47 bcde | 8.53 bcd |
| 1. Atheras | 27.92 abc | 17.67 bcde | 8.67 bcd |
| 1. Agion Oros | 25.00 abcdef | 17.45 bcde | 9.11 abcd |
| 1. Milo Chalkidiki | 24.25 bcdef | 16.58 cde | 8.83 bcd |
| 1. Souvritiki Evrou | 26.17 abcde | 18.50 abcde | 9.33 abcd |
| 1. Boulgariki | 22.00 fg | 14.72 e | 7.33 d |
| 1. Macedonia | 24.00 cdef | 18.08 abcde | 9.25 abcd |
| 1. Milo Corfu | 26.00 abcde | 20.11 abcd | 10.22 abc |
| 1. Milo Cephalonia | 22.78 efg | 20.22 abcd | 9.89 abcd |
| 1. Imvros | 19.78 g | 15.72 de | 7.78 cd |
| 1. Trikala Imathias | 22.25 efg | 18.42 abcde | 9.25 abcd |
| 1. Formula F1 | 24.97 abcdef | 20.92 abc | 10.47 ab |
| 1. Eratiras | 24.44 bcdef | 18.42 abcde | 9.11 abcd |
| 1. Lotos | 25.33 abcdef | 18.92 abcde | 9.50 abcd |
| 1. Nikoulas | 25.25 abcdef | 18.39 abcde | 9.22 abcd |
| 1. Evrou | 27.94 ab | 21.83 ab | 10.94 ab |
| 1. Feneou | 28.50 a | 20.50 abcd | 10.25 abc |
| 1. Aspros lotos | 24.67 abcdef | 19.17 abcde | 9.50 abcd |
| 1. Pantaroza | 26.86 abcd | 20.53 abcd | 10.33 abc |
| 1. Karabola | 24.50 bcdef | 18.33 abcde | 9.08 abcd |
| 1. Kardia vodiou | 25.67 abcdef | 19.06 abcde | 9.72 abcd |
| 1. Takas | 25.56 abcdef | 19.81 abcd | 10.36 abc |
| 1. Pastra | 25.17 abcdef | 22.81 a | 11.53 a |
| 1. Milo Serron | 27.50 abcd | 18.67 abcde | 9.84 abcd |
| Average | **24.92** | **18.82** | **9.49** |

* Varieties with the same letter within a column indicate not significant differences, according to Duncan test (a = 0.05).
