# Supplementary material for: Characterization and evaluation of Greek tomato landraces for productivity and fruit quality traits related to sustainable low-input farming systems
Source: Front Plant Sci. 2022 Dec 12;13:994530. doi: 10.3389/fpls.2022.994530 (PMC9791058; doi:10.3389/fpls.2022.994530)
Supplement: Supplementary file 5 [file Table_3.docx]

**Table S3 │** Early fruit yield (yield/plant, number of fruits/plant, and weight/fruit), vigor or depression (%) (in comparison to the pure line “Macedonia”), and stability of performance (x/s) of the landraces.

| **Landrace** | **Early Fruit Yield** | | | | | | | | | |
| --- | --- | --- | --- | --- | --- | --- | --- | --- | --- | --- |
|  | **Yield/Plant (g)** | | | | **Number of Fruits/Plant** | | | **Weight/Fruit (g)** | | |
|  | x | | V/D | x/s | x | V/D | x/s | x | V/D | x/s |
| 1. Filia Lesvou | 963.47 abc* | | 104 | 1.1 | 3.67 cde | 58 | 2.0 | 227.83 bcdef | 147 | 2.0 |
| 1. Atheras | 0.00 d | | 0 | - | 0.00 e | 0 |  | 0.00 i | 0 | - |
| 1. Agion Oros | 717.90 bcd | | 77 | 1.7 | 5.33 bcde | 84 | 2.4 | 138.10 fgh | 89 | 2.7 |
| 1. Milo Chalkidiki | 1013.40 abc | | 109 | 1.8 | 8.00 abc | 126 | 1.5 | 126.20 fgh | 81 | 3.6 |
| 1. Souvritiki Evrou | 606.65 bcd | | 65 | 1.9 | 5.0 bcde | 79 | 2.5 | 121.33 fgh | 78 | 3.9 |
| 1. Boulgariki | 673.10 bcd | | 72 | 2.0 | 4.0 cde | 63 | 2.0 | 163.50 defgh | 105 | 4.1 |
| 1. Macedonia | 928.63 abc | | - | 2.0 | 6.33 bcd | - | 1.7 | 155.03 efgh | - | 2.9 |
| 1. Milo Corfu | 345.61 cd | | 37 | 1.6 | 1.00 de | 16 | 1.8 | 345.61 a | 223 | 1.7 |
| 1. Milo Cephalonia | 244.50 cd | | 26 | 1.1 | 4.33 cde | 68 | 1.7 | 59.77 hi | 39 | 1.8 |
| 1. Imvros | 681.60 bcd | | 73 | 1.5 | 2.00 de | 32 | 1.6 | 336.00 ab | 217 | 1.5 |
| 1. Trikala Imathias | 608.05 bcd | | 65 | 2.5 | 6.25 bcd | 99 | 2.6 | 94.53 ghi | 61 | 2.5 |
| 1. Formula F1 | 1592.50 a | | 171 | 1.7 | 10.40 ab | 164 | 1.5 | 152.77 efgh | 99 | 5.4 |
| 1. Eratiras | 772.90 abcd | | 83 | 1.6 | 5.07 bcde | 80 | 1.4 | 163.90 defgh | 106 | 3.8 |
| 1. Lotos | 1010.43 abc | | 109 | 1.8 | 7.67 abcd | 121 | 1.8 | 134.93 fgh | 87 | 4.0 |
| 1. Nikoulas | 941.63 abc | | 101 | 2.9 | 3.77 cde | 59 | 1.9 | 299.83 abc | 193 | 1.4 |
| 1. Evrou | 578.17 bcd | | 62 | 1.3 | 3.80 cde | 60 | 1.7 | 167.43 defgh | 108 | 2.3 |
| 1. Feneou | 1197.15 ab | | 129 | 1.6 | 6.75 bcd | 107 | 1.5 | 236.93 bcdef | 153 | 2.5 |
| 1. Aspros lotos | 1221.00 ab | | 131 | 2.1 | 12.77 a | 202 | 2.1 | 95.97 ghi | 62 | 2.2 |
| 1. Pantaroza | 1141.05 ab | | 123 | 2.5 | 6.5 bcd | 103 | 1.7 | 187.28 cdefg | 121 | 2.4 |
| 1. Karabola | 479.27 bcd | | 52 | 1.2 | 3.33 cde | 53 | 1.4 | 144.33 efgh | 93 | 8.1 |
| 1. Kardia vodiou | 1132.20 ab | | 122 | 3.3 | 4.25 cde | 67 | 2.4 | 264.35 abcde | 171 | 2.8 |
| 1. Takas | 446.00 bcd | | 48 | 1.8 | 2.7 cde | 43 | 2.8 | 165.19 defgh | 107 | 3.6 |
| 1. Pastra | 894.95 abc | | 96 | 2.7 | 6.0 bcd | 95 | 4.0 | 138.83 efgh | 90 | 5.0 |
| 1. Milo Serron | 897.80 abc | | 97 | 2.7 | 5.5 bcde | 87 | 5.7 | 159.65 defgh | 103 | 3.9 |
| Average | 828.66 | - | | - | 5.23 | - | - | 175.91 | - | - |

* Varieties with the same letter within a column indicate not significant differences, according to Duncan test (a = 0.05).
